# The* J*_2_ evolution model and control technology of the main roadway surrounding rock under superimposed influence of double-coal seam mining

**DOI:** 10.1038/s41598-023-44136-0

**Published:** 2023-10-16

**Authors:** Dongdong Chen, Zijian Li, Shengrong Xie, Zhiqiang Wang, Zaisheng Jiang, Qingbo Jia, Yiyang Wang

**Affiliations:** School of Energy and Mining Engineering, China University of Mining & Technology-Beijing, Beijing, 100083 China

**Keywords:** Coal, Mineralogy

## Abstract

Under double-seam mining, the main roadway surrounding rock is affected by the superposition of the advanced stress of the two-seam coal working faces. The stress superposition mode and degree are of great significance to the width calculation of the protective coal pillar and the determination of the critical control direction of the surrounding rock. This paper uses theoretical analysis, numerical simulation, and site engineering practice to carry out targeted research. The conclusions are as follows: Under different lateral pressure coefficients, the superposition evolution law of maximum principal stress direction of two coal seams with different offsets; Two developmental trends and three types of evolution models of *J*_2_ peak zone (the critical area of the stress increase and deflection changes) under different superimposed loading modes are summarized. Based on the typical asymmetric evolution model of the *J*_2_ peak zone, an asymmetric truss-cable co-anchoring method is proposed aimed at the *J*_2_ critical zone. The field monitoring results show that the main roadway surrounding rock is stable after support when the upper coal seam protective coal pillar is left 80 m, and the lower one is 60 m wide. It is of great reference importance for similar engineering practices.

## Introduction

Coal is the backbone of China’s energy industry, and there is still a significant demand for coal in China's energy industry^1,2^. However, after years of development, domestic coal occurrence has changed significantly. To prevent wasting coal resources to the maximum extent possible and ensure the energy supply and demand, the mining industry has carried on the rich exploration of new mining technology and new method^3,4^. At the same time, the problem that the overlying coal seam has been widely mined out forces the miners to carry out the deep mining of the lying coal seam. However, due to the influence of the upper mined-out area and the remaining coal pillar, it is inevitable to consider the effect of stress superposition of two coal seams when mining the lower coal seam.

Along with mining, gradually using mature gob-side entry-driving technology is an useful technical means to improve the resource collection efficiency of coal under safe mining^5,6^. When applying the technology of digging roadways along the air, the coal pillar’s width to be protected should be reduced as much as possible to guarantee safety; the reasonable design of the coal pillar’s width is the technology’s critical point. Determining the stopping coal pillar should consider many factors of stope comprehensively. According to the characteristics of the key stratum’s stress field, it is the precondition of safe and effective control to grasp the breaking law of overlying strata correctly^7–10^. To realize the effective control of roadway surrounding rock requires not only the stability of the large structure of the stope but also the in-depth analysis of the deformation and failure mechanism of the coal pillar under the influence of mining. A scientific theoretical calculation model is established to analyze the intrinsic correlation between the coal pillar’s destruction and the movement of crucial blocks in overlying strata^11–13^. While conducting the above research, scholars have paid attention to various failure modes of narrow coal pillars and roadway surrounding rocks in engineering practice and conducted targeted research on the stability of support structures under severe ground pressure behaviors. Furthermore, effective measures such as roof cutting and pressure relief are designed to control the surrounding rock’s deflection and maintain roadway stability^14–20^.

When multi-seam mining is carried out, and the seams’ distance is close, the coal seam’s stress fields will inevitably affect each other, which is distinctly different from single-seam mining^21,22^. Some scholars have studied the stress field around coal pillars and the failure state of coal and rock mass and found that when the coal is mined at a short distance. The upper seam is mined out, and the stress field distribution in the lower coal face is mainly affected by vertical stress; the next is horizontal stress^23,24^. The superposition of the coal seam stress field is not only affected by the spacing between two coal seams but also closely related to the arrangement of the coal face (can be roughly divided into two types:“+” type and “−”Type)^25–27^. Some scholars have studied the behaviors of rock pressure, such as rock bursts, which may be caused by complex stress distribution under the condition of multi-seam mining, the control method which can be applied to engineering practice is put forward^28–31^.

On the basis of the above research, some scholars have explored the failure mechanism and control method of the surrounding rock of the roadway in the lower coal seam under the condition of mined-out in the upper coal seam. The rheological deformation of coal and rock mass and the mining disturbance of the working face will affect the stability of the roadway group, and it is convenient to reveal the deformation and failure mechanism of a large section roadway group in a deep mine. The interaction between the support structure and the surrounding rock is also an essential factor that researchers need to consider. Compared with the research on the failure law of the surrounding rock, it is also significant to study the coordinated deformation of the surrounding rock and anchor cable^32^.

The above research is mainly aimed at the mining roadway, for the main roadway affected by the upper seam mined-out analysis is less. It is necessary to study the deformation and failure mechanism of the surrounding rock of the roadway under the condition of goaf in the upper coal seam. There is no study on the *J*_2_ evolution model of roadway surrounding rock under different superposition modes for double seam mining. *J*_2_ considers the failure of rock mass by normal stress and shear stress, and it can be more scientific and comprehensive to characterize the stress evolution pattern of the surrounding rock and point out the key control direction of the roadway. Therefore, this paper’s in-depth study of the mining of close-range double seams, by the impact of superposition loading, under the conditions of different lateral pressure coefficients and protective coal pillar width, the distortion law of principal stress direction, the superimposed loading mode of *J*_2_ and the internal relationship between them are studied. Three kinds of evolution models of *J*_2_ peak area of the main roadway are obtained, and the asymmetric support technology of channel steel anchor cable with bi-directional reinforcement function are put forward. The engineering practice shows that the critical control direction and control technology are effective, which has important reference significance for determining protective coal pillar width and control method of surrounding rock in similar geological conditions.

## Project profile

### Engineering geology

**Figure 1:**
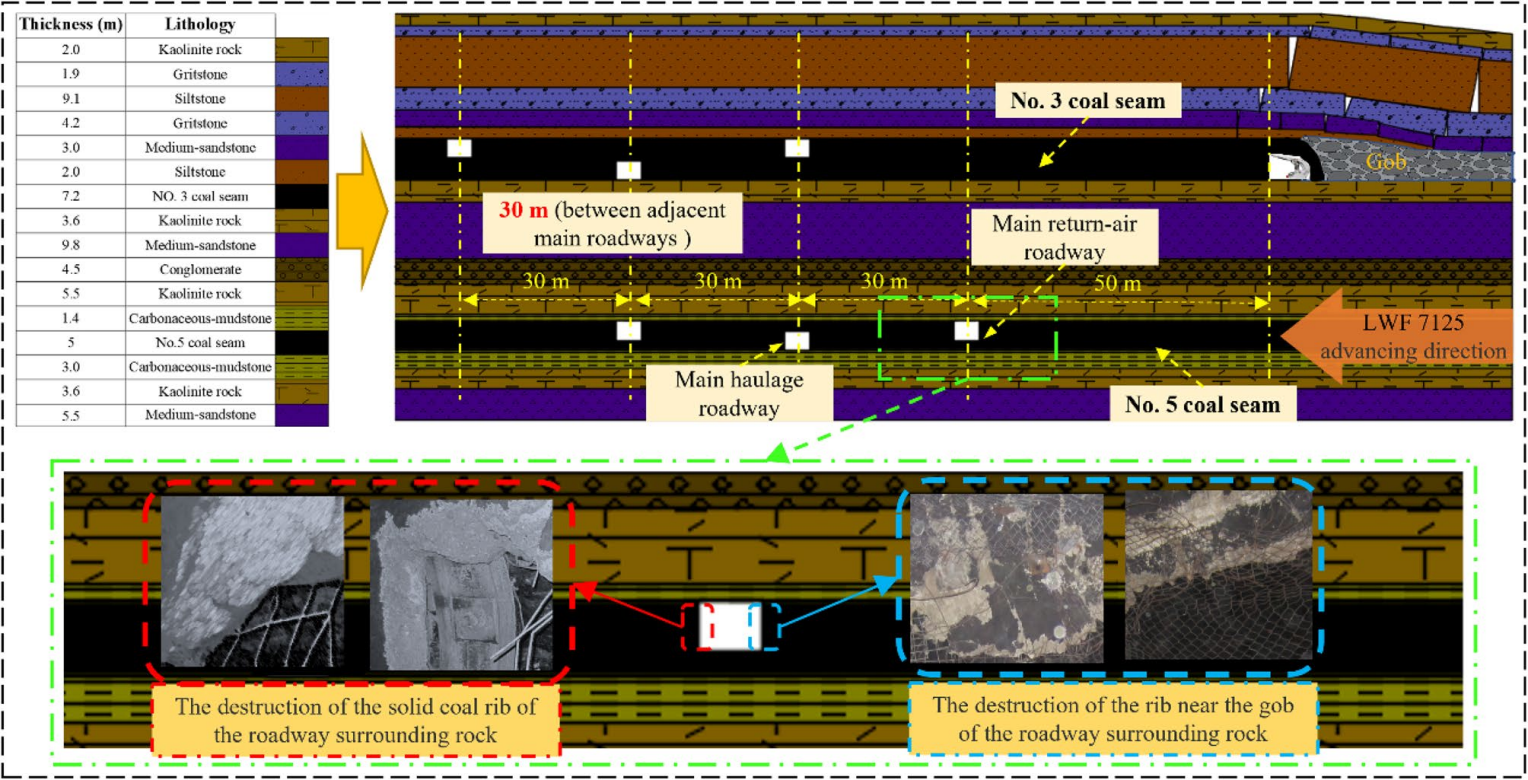
Geological information of rock strata in mining area and the failure of roadway surrounding rock.

This paper is based on a project in Datong, Shanxi province. The project mainly involves mining NO. 3 and NO. 5 two near-horizontal coal seams, the mining area buried depth of about 400 m, and the initial in-situ stress of the research model is about 10 MPa. Figure 1 shows the roadway arrangement, coal seam spacing, and rock stratum distribution in the mining area: No. 3 coal seam is above, the average thickness is about 7.2 m, No. 5 coal seam is below, and the overall thickness is about 5 m. The distance between two coal seams is 25 m, and the distance between adjacent main roadways in two coal seams is 30 m. The upper seam has stopped mining, and mining face LWF7125 is located in the underlying coal seam. The main roadway surrounding rock stability in two coal seams is good and no instability phenomenon occurs, when NO. 3 coal seam mining only. In order to recover the resources as much as possible under the condition of ensuring safety, the reasonable coal pillar width in the NO. 5 coal seam is studied.

### Project profile

**Figure 2:**
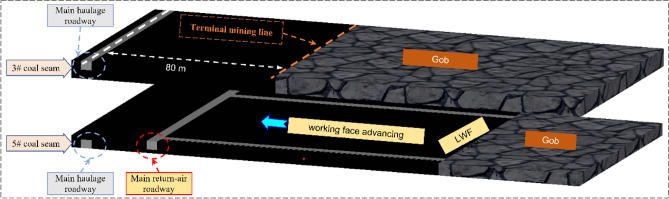
The layout of working surface.

Figure 2 shows the layout of the working face. Under the condition that an 80 m protective coal pillar has been set up in the No. 3 coal seam in the upper part of the mine, due to the existence of the stress superposition situation, the end-mining coal pillar’s width of No. 5 coal seam is usually chosen more significant value (80–120 m) to ensure the stability of roadway group, this also means that more coal resources are not recovered and become less economically efficient. Figure 1 shows the actual situation of the surrounding rock damage. The force of the roadway is extraordinarily complex and uneven due to the superimposed disturbance, and its damage development trend corresponds to the force situation and is not the symmetrical destruction under normal conditions. Therefore, the research on the development pattern of roadway failure direction under the condition of multi-seam mining is helpful for us to design a more reasonable and engineering-specific support scheme and make the support more effective and safer.

## Theoretical analysis

### Rational selection of research indicators

For the study of mining activities under multi-seam geological conditions, the traditional method is usually to analyze the stresses on the coal rock body in terms of vertical stresses. However, for strata affected by superimposed mining activities, relying on a single directional indicator of vertical stress alone for measurement is not scientific and comprehensive enough.

In an effort to gain a deeper insight into the nature of rock deformation and destruction, then clarify the key control direction of the roadway surrounding rock, this paper explores a more comprehensive research index. Following the elastic-plasticity theory^33,34^, the stress at any point in the surrounding rock can be decomposed, as shown in Equation I.

1 $$\sigma_{ij} = \sigma_{m} \delta_{ij} + s_{ij}$$

where *σ*_*m*_ is the spherical tensor component of the stress tensor, *δ*_*ij*_ is the kronecker symbol, and *S*_*ij*_ is the deflection component of the stress tensor.

The action of the two corresponds to different directions of change: the former causes the volume of the object to change under the action of an isotropic force, while the action of the latter causes the force to be uneven, thus causing the deformation of the object, this is also the cause of the formation of the plastic zone in the surrounding rock mass. Among the decomposed stress components, the latter actually influences the deformation and failure of the surrounding rock. In order to analyze the stress state of a point more accurately, it’s also possible to divide its stress states in more detailed terms. However, the results of most stress components will vary with the established coordinate system. This is inconsistent with the actual constant stress state, and the failure threshold of the point will not change with the change of the coordinate system. Based on previous studies, the partial stress tensor’s second invariant (*J*_2_) in the stress component is selected as the index for analysis in this paper. The expression is as follows:

2 $$J_{2} = \frac{1}{6}\left[ {\left( {\sigma_{1} - \sigma_{2} } \right)^{2} + \left( {\sigma_{2} - \sigma_{3} } \right)^{2} + \left( {\sigma_{3} - \sigma_{1} } \right)^{2} } \right]$$

*J*_2_ is independent of the selection of the coordinate system, and it can reflect the deformation and destruction characteristics of the surrounding rock more scientifically because it integrates the damage effect of each principal stress component on the rock mass.

### Solution and damage analysis of stress deformation degree and local stress increase degree under the disturbance of multi-seam mining

**Figure 3:**
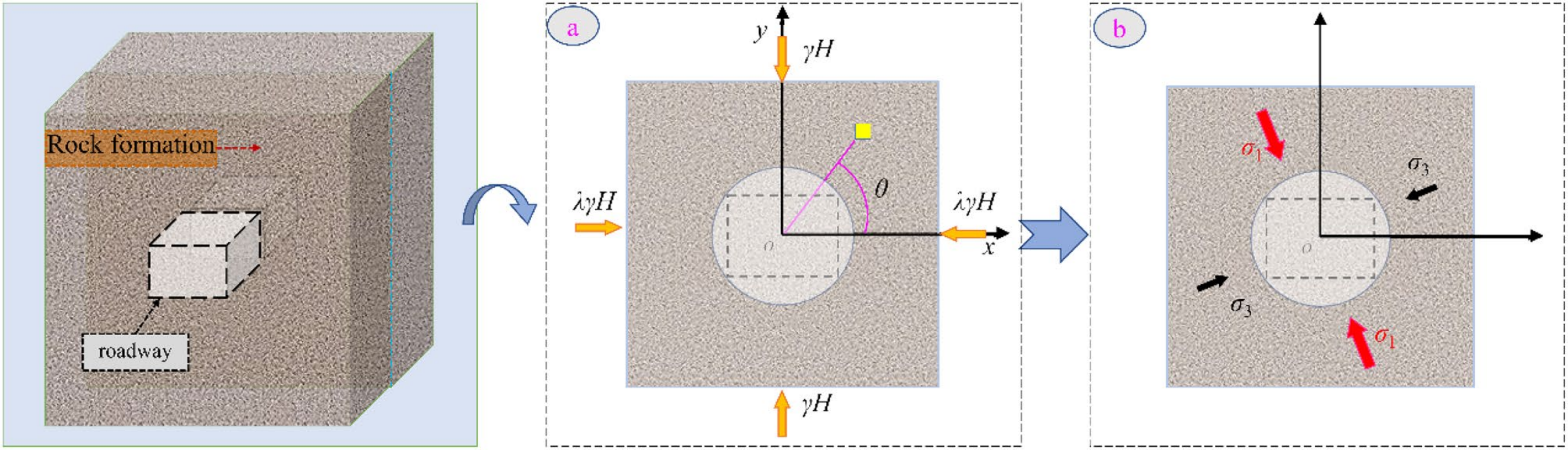
The principle stress state of surrounding rock: (**a**) arbitrary stress field, (**b**) stress field in case of deflection of stress direction and local increase.

As shown in Fig. 3, the magnitude and direction of the principal stresses change under the influence of superimposed mining, and stress increase ande direction change phenomenon occurs in the coal rock body. Deflection and deformation occur in the *J*_2_ peak region, and the damage of the surrounding rock shows a corresponding asymmetric characteristic. The critical failure area can be determined by changing the main stress field and its direction to guide the critical reinforcement direction. The variation of the equivalent components of the principal stresses can be calculated from the following equations ^33^:

3 $$\left\{ {\begin{array}{*{20}c} {\sigma_{1} = \frac{{\sigma_{r} + \sigma_{\theta } }}{2} + \frac{1}{2}\sqrt {\left( {\sigma_{r} - \sigma_{\theta } } \right)^{2} + 4\tau_{r\theta }^{2} } } \\ {\sigma_{3} = \frac{{\sigma_{r} + \sigma_{\theta } }}{2} - \frac{1}{2}\sqrt {\left( {\sigma_{r} - \sigma_{\theta } } \right)^{2} + 4\tau_{r\theta }^{2} } } \\ \end{array} } \right.$$

For the components on the right-hand side of the above equations, the values of the components can be determined by the following formulas.

4 $$\left\{ {\begin{array}{*{20}l} {\sigma_{r} = \left[ {3\left( {\lambda - 1} \right)\cos 2\theta \frac{{a^{4} }}{{R_{\theta }^{4} }} + \left( {1 + \lambda } \right)\left( {1 - \frac{{a^{2} }}{{R_{\theta }^{2} }}} \right) + \left( {\lambda - 1} \right)\cos 2\theta - 4\left( {\lambda - 1} \right)\cos 2\theta \frac{{a^{2} }}{{R_{\theta }^{2} }}} \right]\frac{\gamma H}{2}} \\ {\sigma_{\theta } = \left[ {\left( {1 + \lambda } \right)\left( {1 + \frac{{a^{2} }}{{R_{\theta }^{2} }}} \right) - \cos 2\theta \left( {\lambda - 1} \right) - 3\cos 2\theta \left( {\lambda - 1} \right)\frac{{a^{4} }}{{R_{\theta }^{4} }}} \right]\frac{\gamma H}{2}} \\ {\tau_{r\theta } = \left[ {\sin 2\theta \left( {\lambda - 1} \right) + 2\sin 2\theta \left( {\lambda - 1} \right)\frac{{a^{2} }}{{R_{\theta }^{2} }} - 3\sin 2\theta \left( {\lambda - 1} \right)\frac{{a^{4} }}{{R_{\theta }^{4} }}} \right]\frac{\gamma H}{2}} \\ \end{array} } \right.$$

where $\sigma_{r}$ h is radial stress; $\sigma_{\theta }$ is circumferential stress; $\tau_{r\theta }$ is shear stress; *γ* is the weight density of the rock; *H* is the depth of the road; *λ* is the coefficient of lateral pressure; a is the radius of the circular roadway. The polar coordinates of any point are set to ($\theta$, $R_{\theta }$).

Put Formula 3 into Formula 2 to get Formula 5, which is the expression of *J*_2_ in polar coordinates:

5 $$J_{2} = \frac{1}{3}\left( {\sigma_{r}^{2} + \sigma_{\theta }^{2} - \sigma_{r} \sigma_{\theta } } \right) + \tau_{r\theta }^{2}$$

By inserting Formula 4 into Formula 5, it get the expression of *J*_2_:

6 $$J_{2} = \left\{ \begin{gathered} 4\left( {\lambda^{2} - \lambda + 1} \right) - \left[ {22\left( {\lambda^{2} - 1} \right)\cos 2\theta - 12\left( {\lambda - 1} \right)^{2} + 12\left( {\lambda - 1} \right)^{2} \cos^{2} 2\theta } \right]\frac{{a^{2} }}{{R_{\theta }^{2} }} \hfill \\ + \left[ {\left( {15\lambda - 6\lambda^{2} - 3} \right) + 12\left( {\lambda^{2} - 1} \right)\cos 2\theta + 40\left( {\lambda - 1} \right)^{2} \cos^{2} 2\theta } \right]\frac{{a^{4} }}{{R_{\theta }^{4} }} \hfill \\ - \left[ {18\left( {\lambda^{2} - 1} \right)\cos 2\theta + 36\left( {\lambda - 1} \right)^{2} } \right]\frac{{a^{6} }}{{R_{\theta }^{6} }} + 27\left( {\lambda - 1} \right)^{2} \frac{{a^{8} }}{{R_{\theta }^{8} }} \hfill \\ \end{gathered} \right\}\frac{{\gamma^{2} H^{2} }}{12}$$

7 $$\left\{ \begin{gathered} \frac{{\left( {1 - 3\tan^{2} (45^\circ - \frac{\varphi }{2})} \right)}}{{\left( {1 + \tan^{2} (45^\circ - \frac{\varphi }{2})} \right)}}\left[ \begin{gathered} \frac{\gamma H}{4}\left( {2\left( {\lambda - 1} \right) - 4\left( {\lambda - 1} \right)\cos 2\theta \frac{{a^{2} }}{{R_{\theta }^{2} }}} \right) \hfill \\ + \frac{{\tan^{2} (45^\circ - \frac{\varphi }{2})\left( {2C\tan (45^\circ + \frac{\varphi }{2}) - 6C\tan (45^\circ + \frac{\varphi }{2})\tan^{2} (45^\circ - \frac{\varphi }{2})} \right)}}{{1 - 3\tan^{2} (45^\circ - \frac{\varphi }{2})}} \hfill \\ \end{gathered} \right]^{2} \hfill \\ = \left[ \begin{gathered} 36\left( {\lambda - 1} \right)^{2} \frac{{a^{8} }}{{R_{\theta }^{8} }} - \left( {48\left( {\lambda - 1} \right)^{2} + 24\left( {\lambda^{2} - 1} \right)\cos 2\theta } \right)\frac{{a^{6} }}{{R_{\theta }^{6} }} \hfill \\ + \left( {48\left( {\lambda - 1} \right)^{2} \cos^{2} 2\theta + 16\left( {\lambda^{2} - 1} \right)\cos 2\theta + 24\lambda - 4\lambda^{2} - 4} \right)\frac{{a^{4} }}{{R_{\theta }^{4} }} \hfill \\ - \left( {8\left( {\lambda^{2} - 1} \right)\cos 2\theta - 16\left( {\lambda - 1} \right)^{2} } \right)\frac{{a^{2} }}{{R_{\theta }^{2} }} + 4\left( {\lambda - 1} \right)^{2} \hfill \\ \end{gathered} \right]\frac{{\gamma^{2} H^{2} }}{16} + \frac{{4C^{2} \tan^{2} (45^\circ + \frac{\varphi }{2})\tan^{6} (45^\circ - \frac{\varphi }{2})}}{{1 - 3\tan^{2} (45^\circ - \frac{\varphi }{2})}} \hfill \\ \end{gathered} \right\}$$

**Figure 4:**
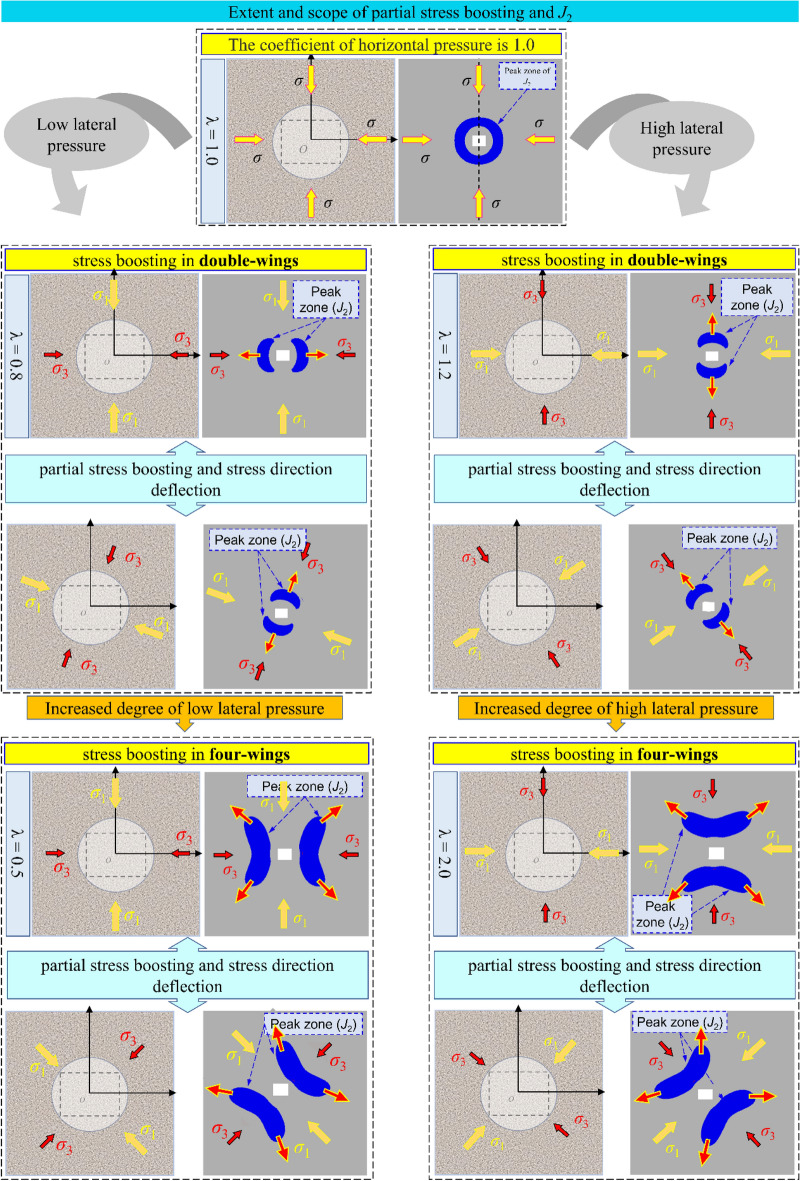
The evolution model of *J*_2_ peak zone of surrounding rock under different coefficients of horizontal pressure and stress field loading direction.

It is available to analyze the evolution^35,36^ of principal stresses and *J*_2_ in the surrounding rock under different variable conditions combined with the above equations. As in Fig. 4, the Intuitive evolution process of* J*_2_ under the conditions of with conditions of stress increase and principal stress deflection or not can be obtained by combining the numerical simulation.

The area and deflection of the *J*_2_ peak region (key area) varies with the different scales of stress increase and directional deflection, which indicate the critical failure development area and direction of the roadway surrounding rock. When the scale of stress increase and deflection changes is small, the *J*_2_ peak zone of the roadway surrounding rock develops in double-wings pattern (mainly in the two symmetrical directions). When the scale of stress increase and deflection changes becomes more serious, the *J*_2_ peak zone develops in a four-wings pattern (the destruction area tends to evolve from four directions into the deep part). This suggests that the development of the roadway damage is asymmetric under the stress increase and deflection change, and in the process of the damage continues to deepen, the *J*_2_ critical zone will show the two possible evolution modes mentioned above, which is an essential guideline for designing a more reasonable roadway support scheme.

## Numerical simulation

Numerical simulation is an effective means to visually analyze the change in the surrounding rock stress state and the degree of damage. This is benefical to analyze the damage development pattern of the roadway and to determine its critical reinforcement location.

The numerical model is applied to explore the following three aspects: (1) under different lateral pressure coefficients when the misalignment distance between coal seams is different (That is, the lower coal seam terminal mining line of the working face is ahead of the upper coal seam terminal mining line by 50 m, 30 m, 10 m and behind the upper coal seam stopping line by 10 m, 30 m respectively), the evolution law of principal stress ahead of the working face. (2) The distribution and evolutionary development pattern of the *J*_2_ peak zone when left 80 m coal pillar in the upper coal seam and set different protective coal pillar width in the lower coal seam. Parameters of the coal rock body used in the simulation are referenced in Table 1.

**Table 1:** The parameters of rock mass of LWF 7125.

Lithology	Density (kg∙m^-3^)	Elastic modulus (GPa)	Friction (°)	Cohesion (MPa)	Tensile strength (MPa)
Gritstone	2650	17.43	32	3.16	2.42
Siltstone	2602	14.59	35	3.07	2.17
3# coal seam	1400	3.79	18	1.49	1.21
Conglomerate	2660	18.08	34	3.5	3.13
5# coal seam	1400	3.80	18	1.49	1.21
Carbonaceous-mudstone	2200	14.77	29	2.94	2.22
Kaolinite rock	2570	17.00	38	5.18	2.78
Medium-sandstone	2557	12.69	38	6.26	3.63

### The pattern of principal stress changes ahead of the working face in superimposed mining

In multi-seam mining, the different position relations of the terminal mining line of two coal seams also represent different superposition types of overburden rock structure: external-offset, that is, the lower coal seam end-mining line lies below the upper coal seam mined-out area; The coal seams end-mining lines overlap, and the internal-offset means that the lower coal seam end-mining lines lie beneath the upper one’s solid coal.

Under the different spatial positions of the protective coal pillar in the two coal seams, the superposition effect of the stress field with different properties will naturally appear. When the final selected width of the NO. 5 coal seam protective coal pillar is shorter than the upper one, the NO. 5 coal seam’s working face will experience the influence of the superposition of the above three overburden rock structure states, respectively.

The stress components at fixed points before each working face are obtained by numerical calculation under different overburdened rock structure positions. The angle of deflection of the vertical direction line to the maximum principal stress direction line is recorded as α, which can be calculated (α is "+" when the deflection trajectory is counterclockwise; otherwise, it is "−", as well as the absolute value of α is less than 90°). The figure shows the principal stress state ahead of the coal seam working faces and the deflection law when the offset between two coal seams working faces is different. To get a more accurate grasp of the deflection law of the principal stress direction under multi-seam mining conditions, a reference group with lateral pressure coefficients of 0.8 and 1.8 is used for comparison study.

**Figure 5:**
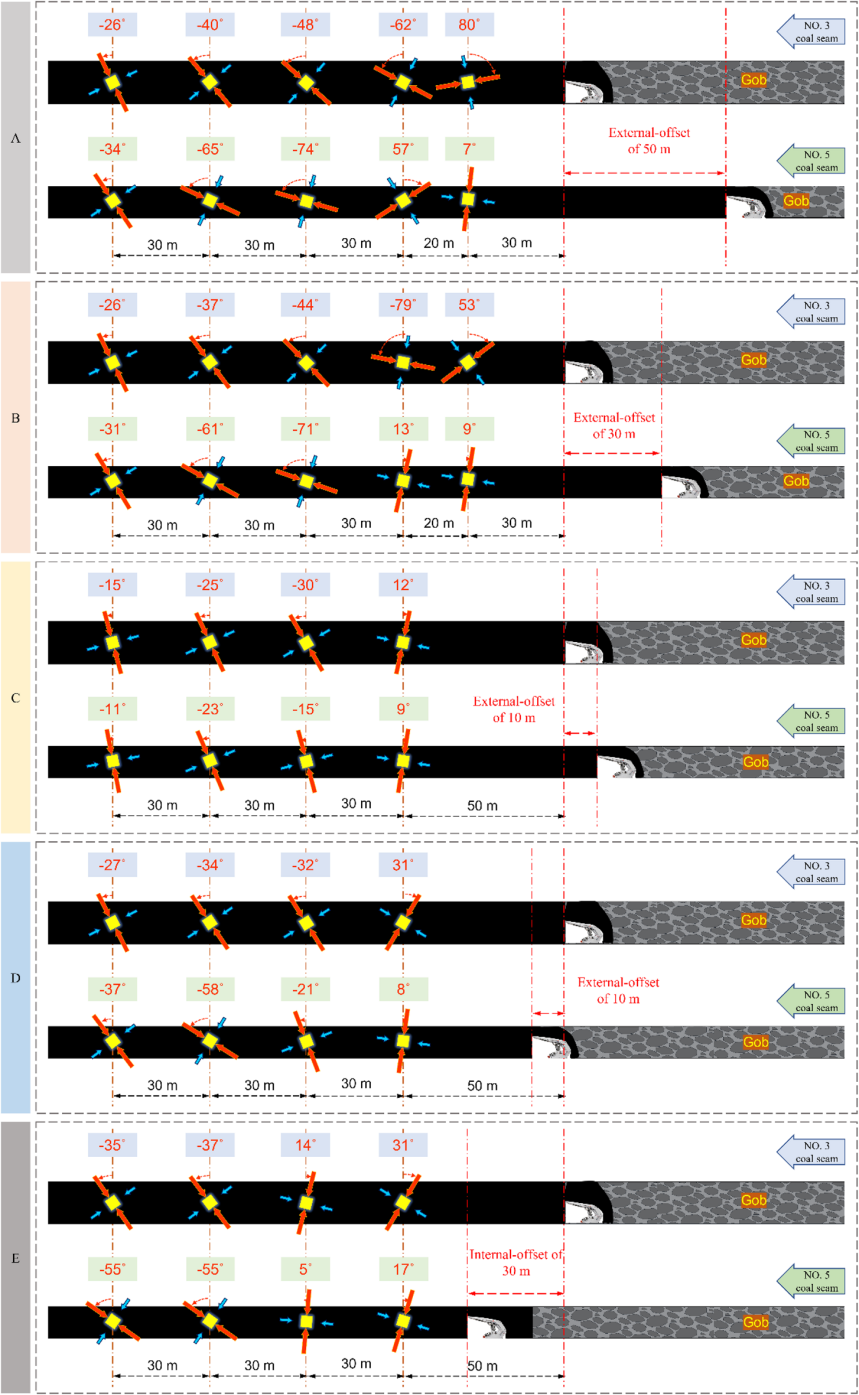
Variation of principal stress direction when the two coal seams working faces has different offset that lateral pressure coefficient is 0.8.

**Figure 6:**
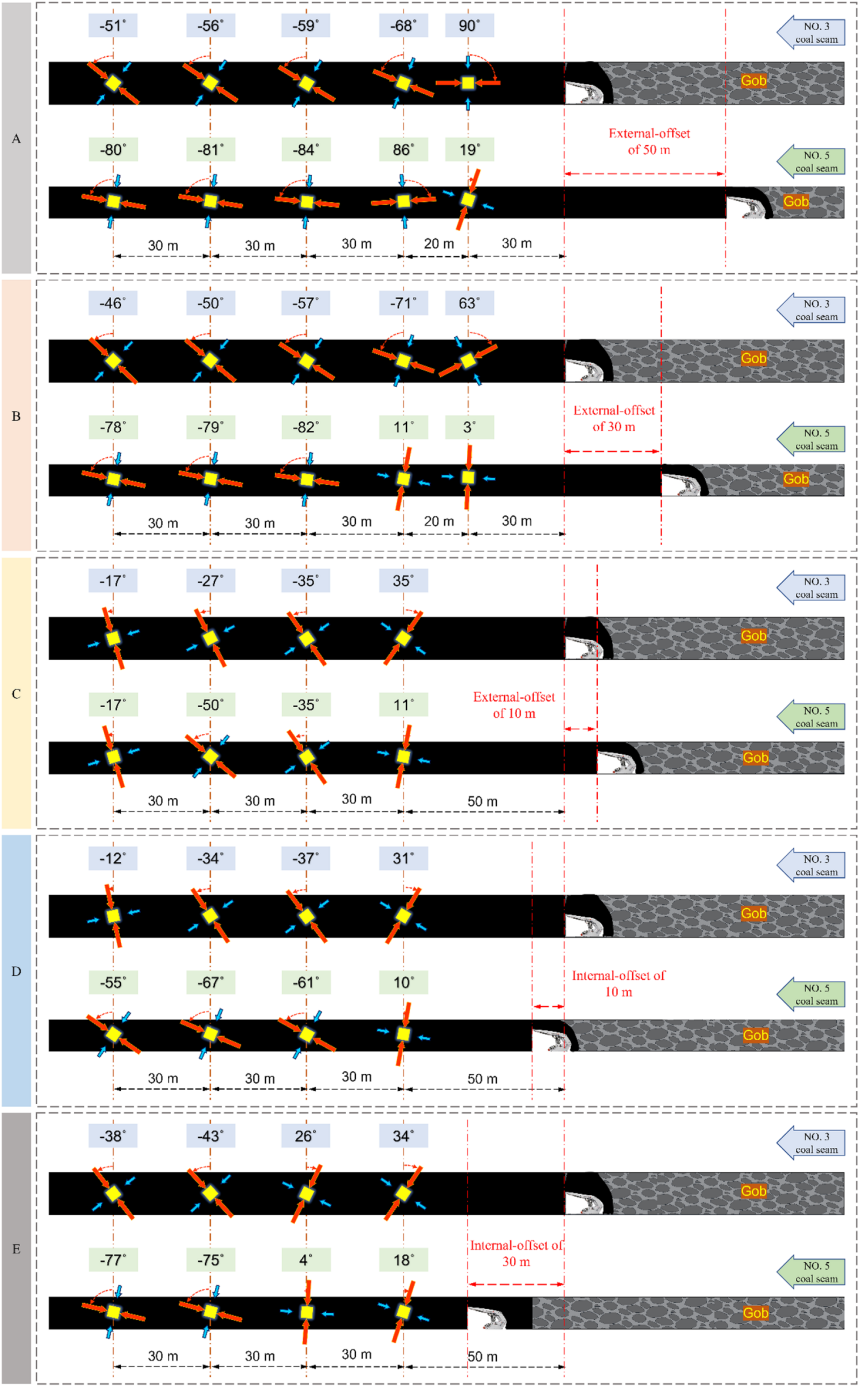
Variation of principal stress direction when the two coal seam working faces has different offset that lateral pressure coefficient is 1.2.

**Figure 7:**
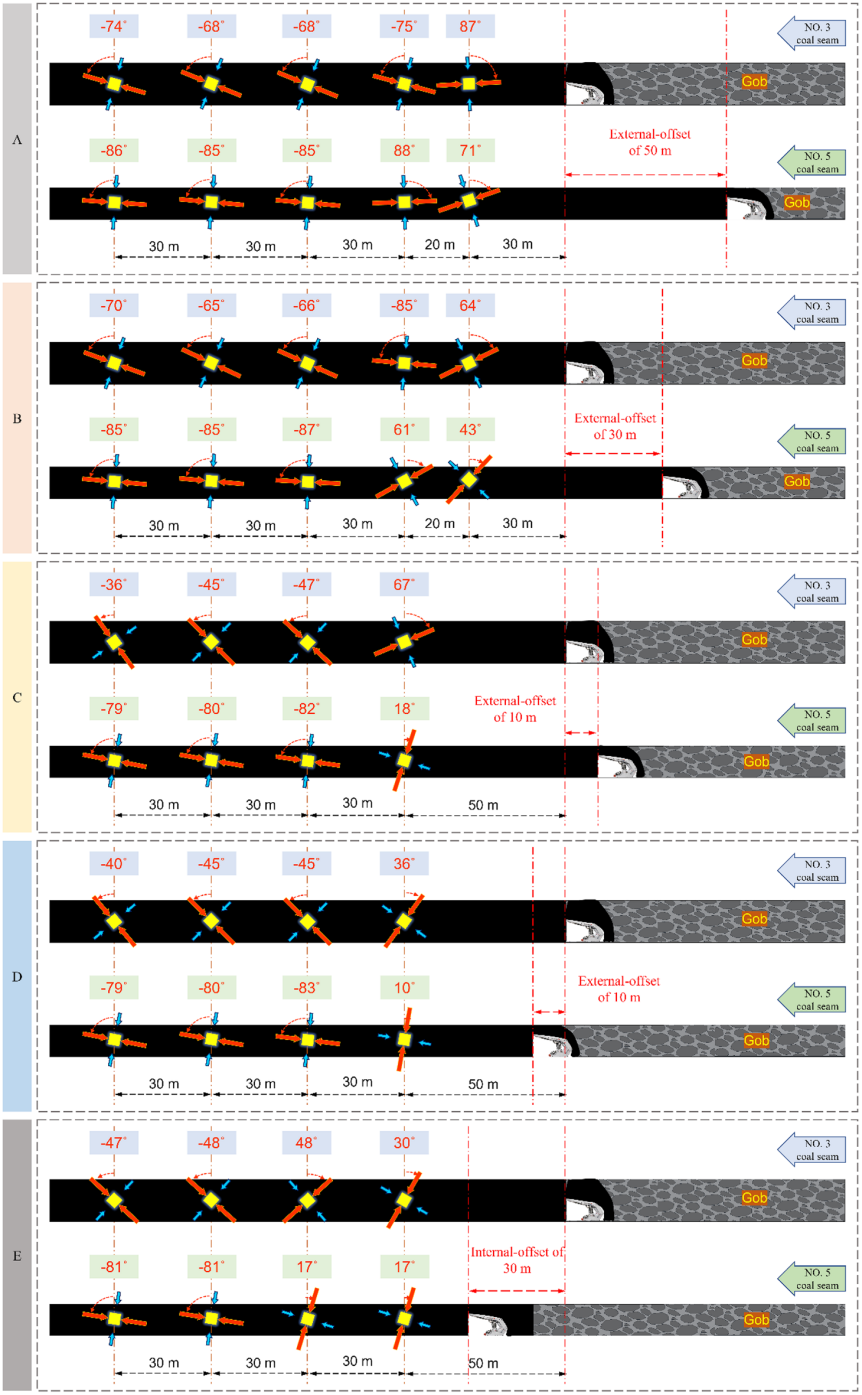
Variation of principal stress direction when the two coal seam working faces has different offset that lateral pressure coefficient is 1.8.

**Figure 8:**
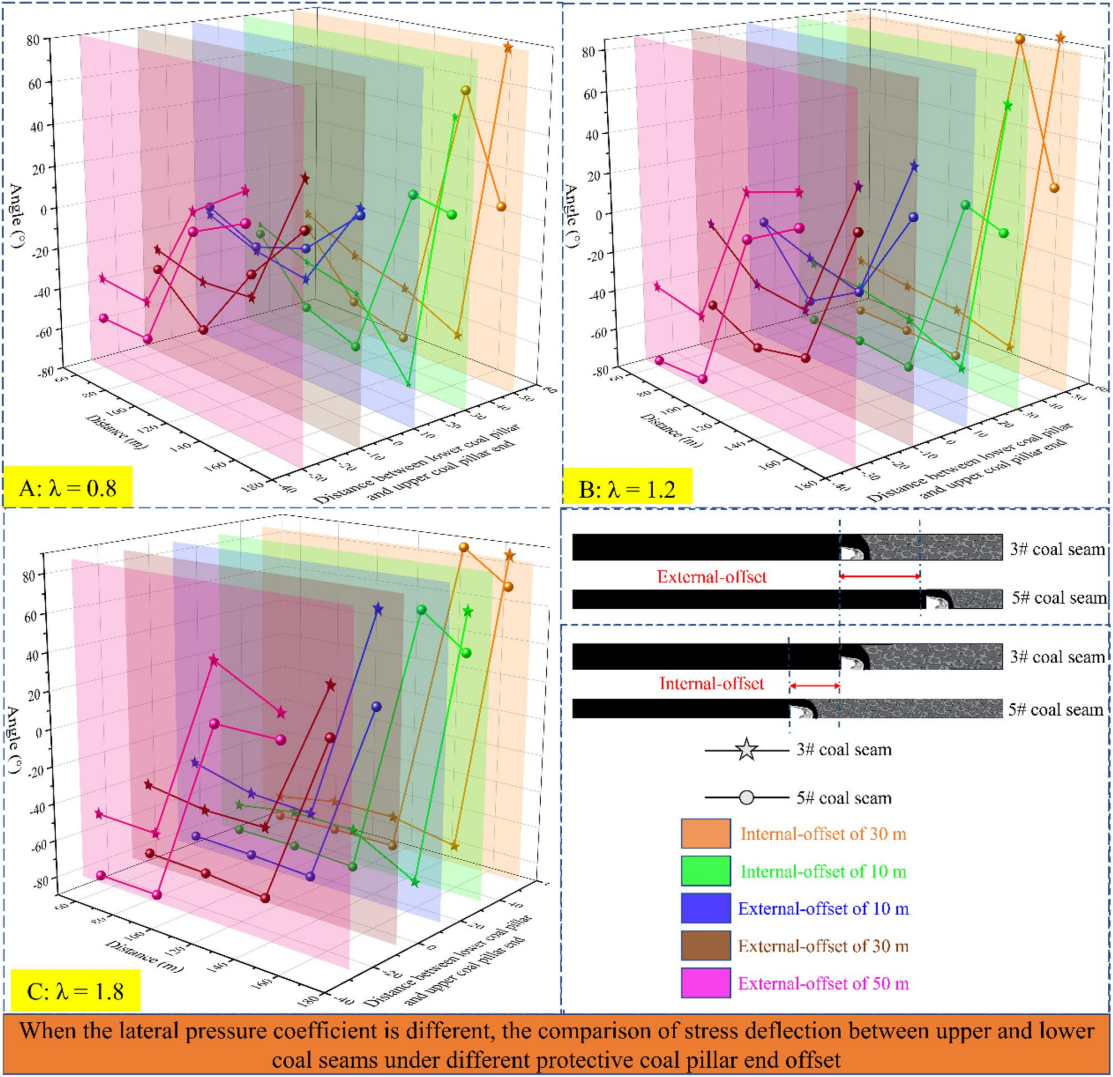
The deflection of principal stress in two coal seams under different lateral pressure coefficients.

As shown in Figs. 5, 6, 7 and 8 above, the deflection law of the maximum principal stress direction is obtained as follows:

1. When the calculation point is near the mined-out area, the maximum principal stress direction deviates to the side of the mined-out area; when it is far away from the mined-out area, it changes to the side of solid coal.
2. The critical region of lower coal seam α is more ahead than the upper. The α critical area of the two coal seams is close to each other when the deflection of maximum principal stress causes the value of α to convert from “ + ” to “−” and gradually deep into the solid coal.
3. The critical region of α when the location relationship of two coal seams is external-offset is different from the internal-offset. The critical direction of α is horizontal for a significant degree of external-offset and vertical for approximate overlap and internal-offset. The roof and floor support and maintenance are complicated, so it is reasonable to place the roadway at a position where the directional line of α is approximately vertical.
4. As the lateral pressure coefficient increase, the change rate of α significantly increases when the two coal seams working faces has the same offset distance condition. The difference in α between the two coal seams at the same position increases, and the principal stress’ deflections is more remarkable.

### The superposition evolution law of ***J***_2_ stress field under different protective coal pillar’s widths of underlying coal seam

**Figure 9:**
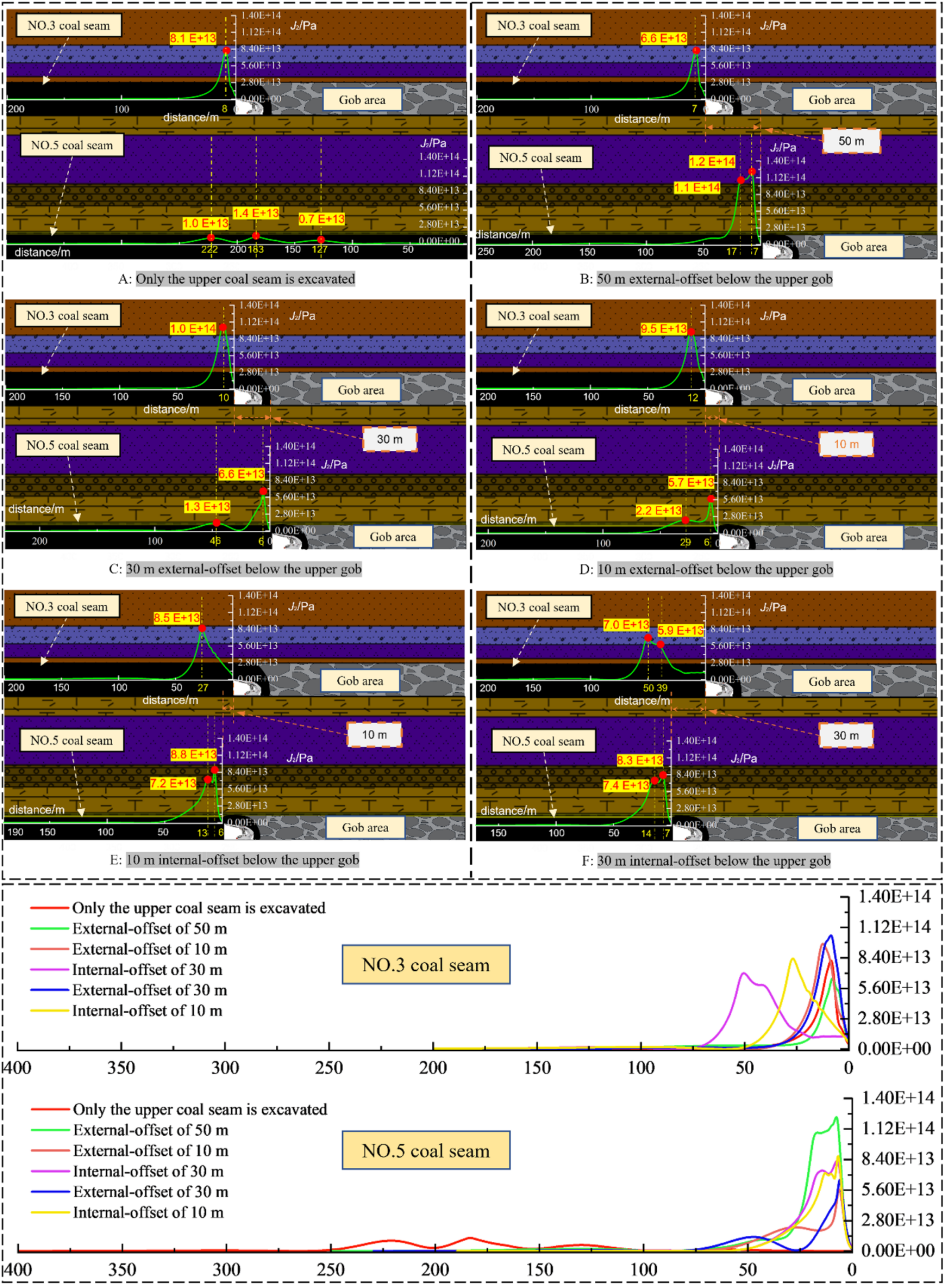
The magnitude and distribution of *J*_2_ stress field ahead of the working face under various dislocation relationships in the two coal seam working faces.

The deviatoric stress tensor is the stress tensor component at a point in the body during plastic deformation, which changes with the change of coordinates, but its stress tensor invariant is fixed. Therefore, the invariant of the deviator stress tensor can indicate the exact nature of plastic damage of the object. Taking *J*_2_ as the Research Index, the evolution law of each coal face’s advance abutment stress field under different offsets between two coal seams is researched when the main roadway is not excavated, as shown in Fig. 9.

The conclusion is as follows:

When the upper seam set 80 m protective coal pillar:

1. In the process of No. 5 coal seam not mined to the upper and lower working face position relationship of external-offset 50 m, the *J*_2_ peak value in No. 3 coal seam first slightly decreases due to the influence of mining. Then the peak value increases and then decreases with the further mining of No.5 coal seam, and the maximum load increase factor *L* = 1.51.
2. In the process of No. 5 coal seam not mined to the upper and lower working face position relationship of external-offset 50 m, the peak stress of *J*_2_ presents double peak value characteristics, and the load-increasing coefficient *L* = 8.57. The *J*_2_ peak value in NO. 5 coal seam decreases firstly, and the ratio of the peak value of *J*_2_ between external-offset 10 m and external-offset 50 m is 0.47, the ratio of *J*_2_ peak value at internal-offset 30 m to 10m external-offset is *Rs* = 1.45.
3. With the mining of NO. 5 coal seam’s working face, there's a remarkable variance in the distance between the two coal seam *J*_2_ peak value lead the working face. The NO. 5 coal seam *J*_2_ peak depth is kept at 6–7 m, and the NO. 3 coal seam *J*_2_ peak value gradually penetrates the deep solid coal area, from 7 to 50 m ahead of the working face, increasing by 614%.

**Figure 10:**
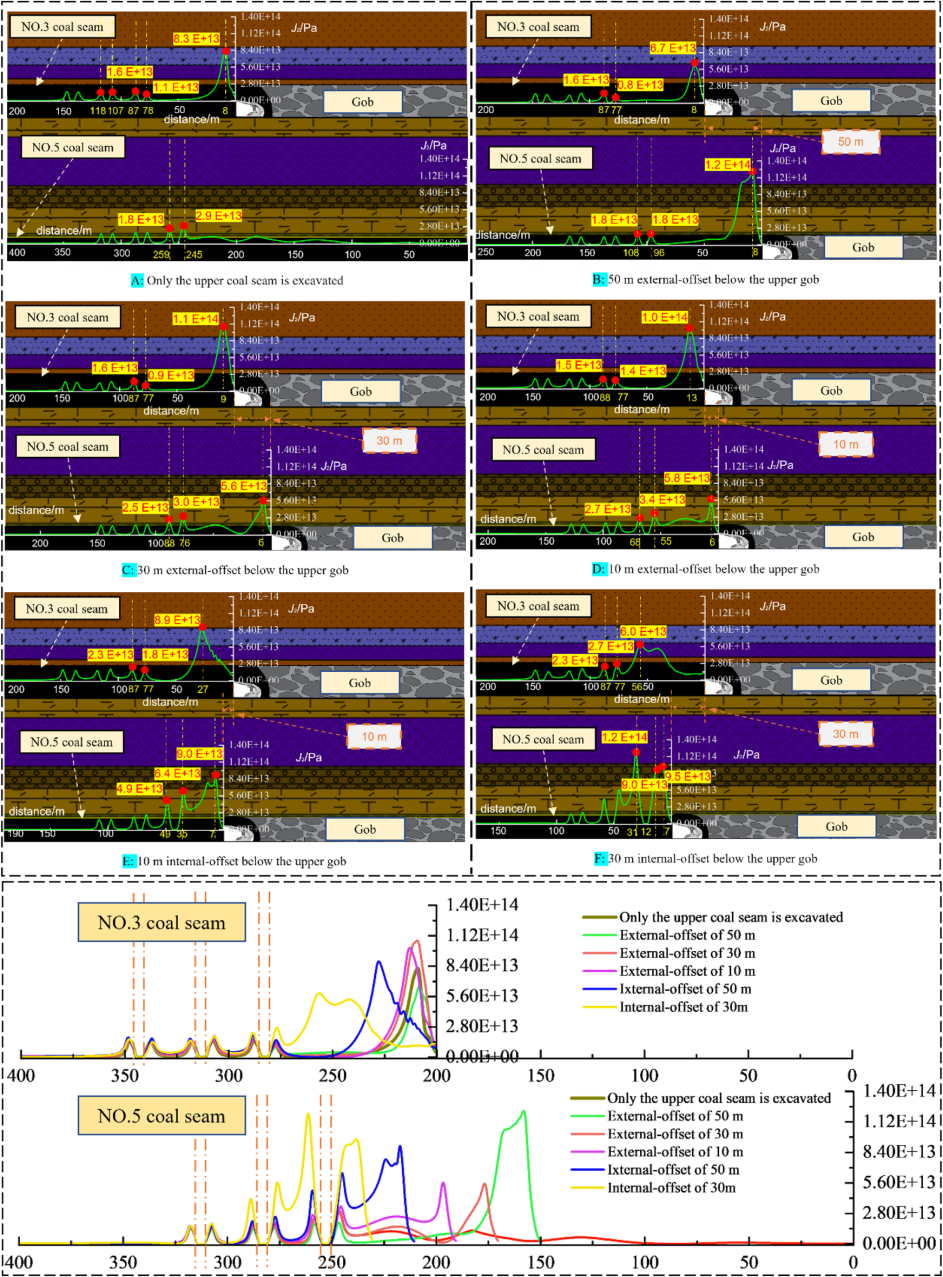
The magnitude and distributions of *J*_2_ stress field ahead of the working face under various dislocation relationships in the two coal seam working faces.

After excavating the main roadway, Fig. 10 shows the curve of the working face’s *J*_2_ under different two coal seams’ stagger distances.

When the protective coal pillar’s width of NO. 3 coal seam is 80 m:

1. The evolution pattern of the *J*_2_ peak value in NO. 3 coal seam is consistent with that one when the main roadways unexcavated. However, after excavating the lower coal seam, the peak value of *J*_2_ on both sides of the lower main return-air roadway decreased slightly at first, then it increased continuously with the advance of the working face. The maximum load-increasing coefficient of the *J*_2_ peak value near the mined-out area is *L* = 3.37, and that one near the solid coal is *L* = 1.53. Before the working face advances to an internal-offset 30 m, the peak value of *J*_2_ near the mined-out area is smaller than that one near the solid coal. The opposite is true after the two coal seams’ working faces are internal-offset 30 m.
2. The* J*_2_ peak value in NO. 5 seam increases gradually when the working faces’ spatial location relationships is external-offset; the ratio of the *J*_2_ peak value in external-offset 50 m to that in external offset 10 m is *Rs* = 0.48, and the peak value of *J*_2_ increases gradually after the continuous advance of the face changes to the internal-offset, the maximum loading coefficient *L* = 2.07. The peak value of *J*_2_ on both sides of the main return-air roadway in NO. 5 coal seam is more significant than that in NO. 3 coal seam, and the general evolution law is similar to the upper one.
3. The evolution of roadway surrounding rock’s *J*_2_ presents a typical asymmetric stress increase and deflection changes effect. Following the decrease of the protective coal pillar’s width in NO. 5 coal seam, the *J*_2_ peak value near the main return airway minied-out area is obviously higher than the solid coal side. This phenomenon shows that the stress field around the roadway has obvious asymmetry, so it is essential to adopt directional support in the surrounding rock's vulnerable area.

The evolution of *J*_2_ can feedback the elastic–plastic state of the object. It can better reflect the intrinsic nature of plastic distortion. The *J*_2_ peak zone shows the area is in the stage of destruction development. Under the mining influence, especially the complex disturbance of close-distance double seams mining, it is easy to cause the stress increase and deflection changes effect, then lead to the asymmetric evolution of *J*_2_ peak zone. It is of paramount importance to explore the evolution patterns of the surrounding rock’s *J*_2_ peak zone for studying the failure mechanism of roadway surrounding rock and developing key reinforcement technology of roadway. Under the condition of stopping mining pillar 80 m in NO. 3 coal seam, with mining influence the *J*_2_ peak value zone's development law of roadway surrounding rock when the protective coal pillar's width is different in NO. 5 coal seam is studied, as shown in the following diagram.

**Figure 11:**
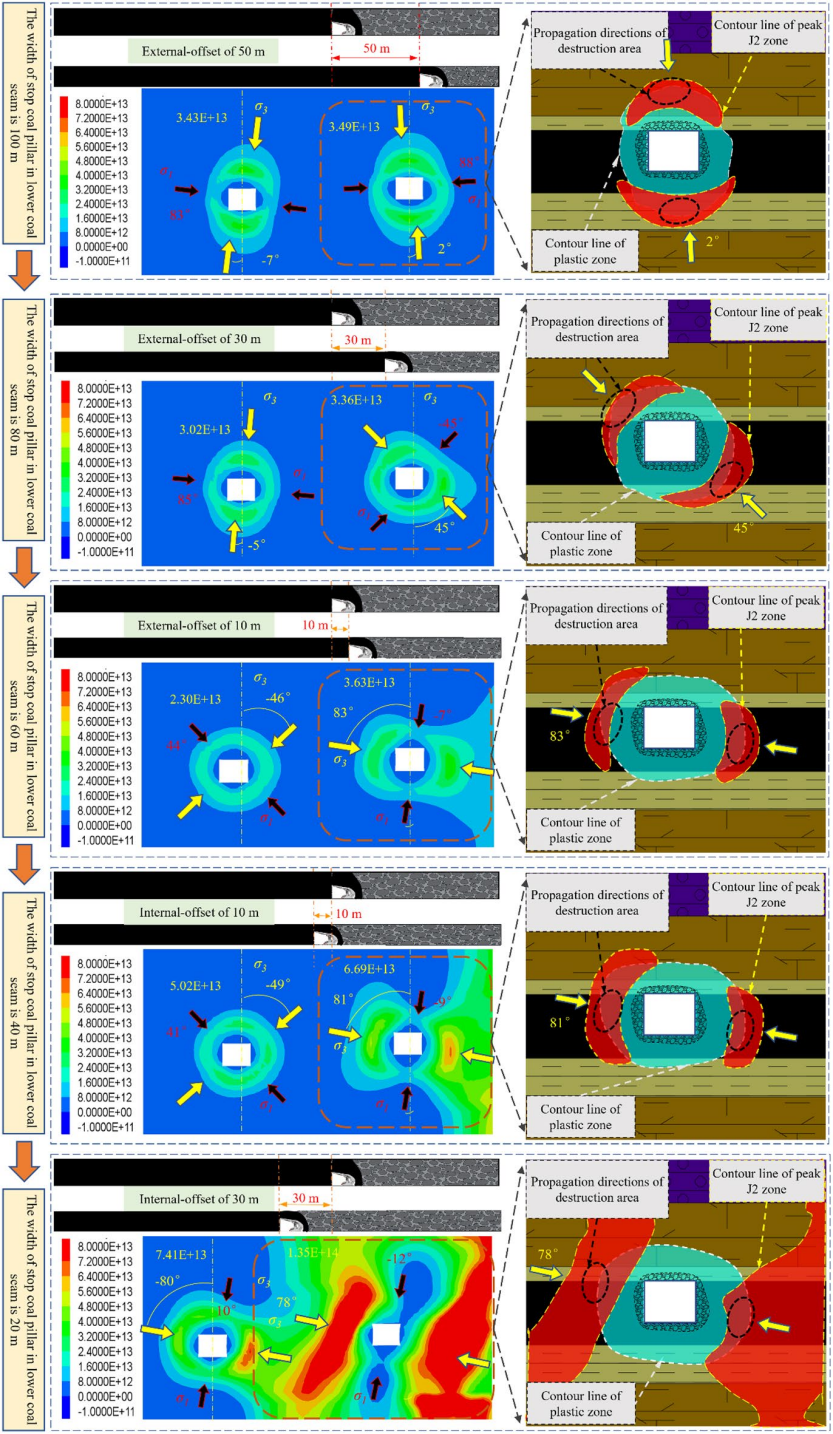
Distribution and development of *J*_2_ peak zone when the two coal seams working faces are under different spatial location relationships.

**Figure 12:**
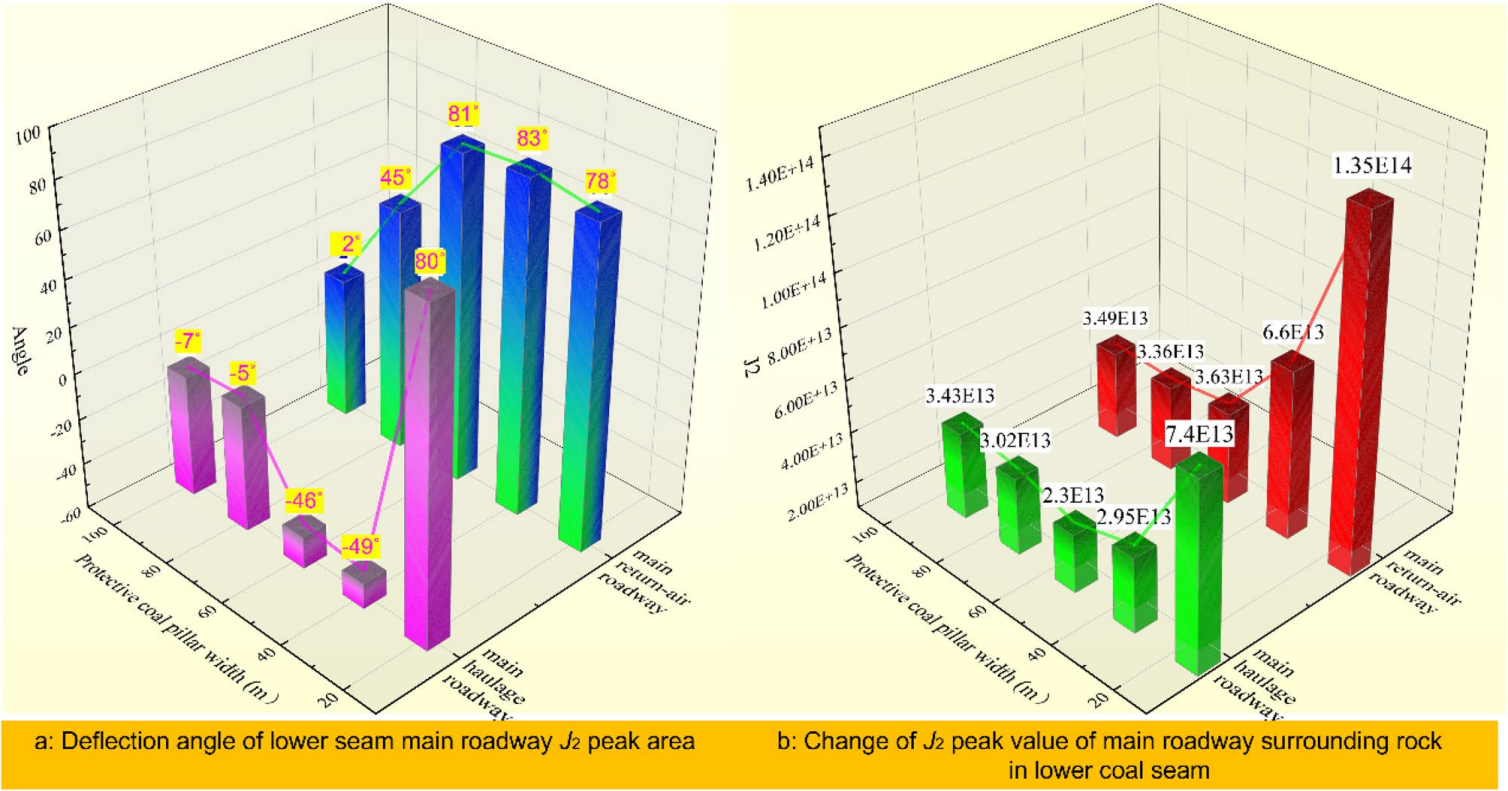
The evolution situation of *J*_2_ in the main roadway (**a**): deflection of *J*_2_ peak zone; (**b**): magnitude of peak stress of *J*_2_.

Figures 11 and 12 show that during mining of NO. 5 coal seam:

1. From the developmental morphology of *J*_2_ peak zone: The *J*_2_ peak zone appears to enlarge gradually with mining, and there is a tendency of gradually surrounding the roadway; And the deflection phenomenon of the *J*_2_ peak zone is evident during the development, which gradually deflects from the roof-floor area to the ribs, and then develops to the surrounding rock’s depth. The growth trend of the plastic area has the same significant directivity. The plastic zone’s outlines passed through the central region of the peak area of *J*_2_.
2. According to the *J*_2_ peak value evolution rule, the *J*_2_ peak value shows an increasing trend as the protective coal pillar’s width decreases. When the protective coal pillar's width is decreased from 100 to 60m, the *J*_2_ peak value load-increasing coefficient is* L* = 1.04, but when the protective coal pillar’s width is from 60 to 40 m, *L* = 1.84, and when the protective coal pillar's width is from 40 to 20 m, *L* = 1.95. This phenomenon indicates that the fluctuation of the peak value of *J*_2_ is not apparent when the stopping pillar is more significant than 60 m in the NO. 5 coal seam. However, when the width is shorter than 60 m, the *J*_2_ peak value increases sharply, the surrounding rock becomes more easily destroyed, and the support of the main roadway is complicated.
3. From the principal stress deflection: the principal stress deflection characteristic of the main return-air roadway’s surrounding rock in the NO. 5 coal seam is prominent, and the changes of the minimum principal stress’ direction correspond to the *J*_2_ peak value zone’s deflection, which is consistent with the result of the theoretical derivation. As the protective coal pillar’s width decreases, the minimum principal stress develops from approximate vertical to approximate horizontal, from pointing to the roof-floor area of the roadway to pointing to both ribs.
4. While the protective coal pillar’s width is 60 m in NO. 5 coal seam, the minimum principal stress direction in the surrounding rock is about horizontal. Currently, the *J*_2_ peak zone is asymmetrically distributed at the two ribs of the roadway. The simulation results are the same as the actual location of the damage to the roadway with on-site conditions and uncovered the damaged organism of the roadway surrounding rock. This is significant for preventing and controlling surrounding rock failure and rationally applying asymmetric control technology.

## Roadway stability control technology

### Principle of asymmetric cooperative anchoring method

The surrounding rock affected by mining will form a key area of stress increase and deflection changes, the *J*_2_ peak zone. It indicates the foreboding of the failure of the surrounding rock and leads the evolution process of the continuous development of the surrounding rock’s failure. On this basis, it puts forward the principle of non-symmetric orientation cooperative anchoring of the surrounding rock. That is, based on the evolution characteristics of stress increase and deflection changes, the critical areas of the targeted high-prestressed anchor cable reinforcement to design a more scientific and reasonable support program to ensure the roadway’s stability.

**Figure 13:**
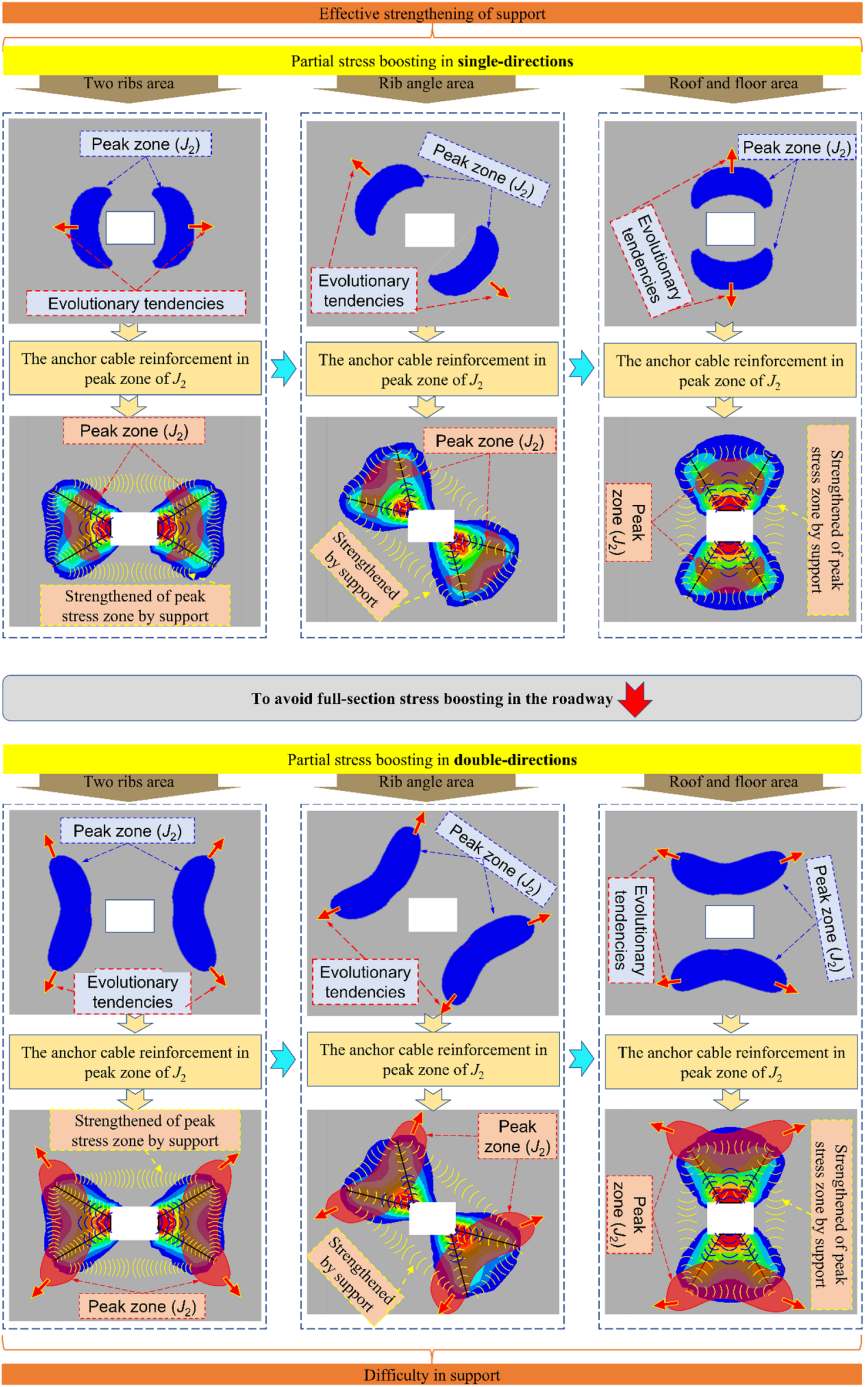
Principle of non-symmetric orientation cooperative anchoring.

The results of the above study suggest that, with the effect of multi-seam mining, there are three kinds of critical areas of stress increase and deflection changes in the roadway: roof-floor area, two ribs area, ribs-roof-floor transition area (2 kinds: upper left-lower right corner area, lower left-upper right corner area). The *J*_2_ peak value area of the main roadway is the critical area of stress increase and deflection changes, the key area of directional cooperative anchoring. The roadway surrounding the rock’s corresponding reinforcement and anchoring schemes are put forward for three critical areas with various scales of stress increase and deflection changes. Figure 13 shows the anchoring principle.

When the scale of the stress increase and deflection changes is low, it develops to the depth of surrounding rock in a double-wings pattern. Therefore, the reinforcement anchor cable is added to the surrounding rock’s *J*_2_ peak value area so that the stress field formed by the reinforcement anchor cable can effectively cover the critical area of stress increase and deflection changes. Due to the anchoring action of the anchor cable, the high prestress and high shear failure resistance provided by the anchor cable can effectively enhance the overall bearing surrounding rock’s capacity.

When the scale of the stress increase and deflection changes is higher, it begins to develop to the surrounding rock’s depth by a four-wings pattern. At this time, the stress field of reinforcement support is challenging to effectively cover the failure trend in many directions; that is, stress increases and deflection changes are out of control. It also means that the surrounding rock is severely damaged. We must avoid this as much as possible in this situation’s actual project.

### Design of support scheme

**Figure 14:**
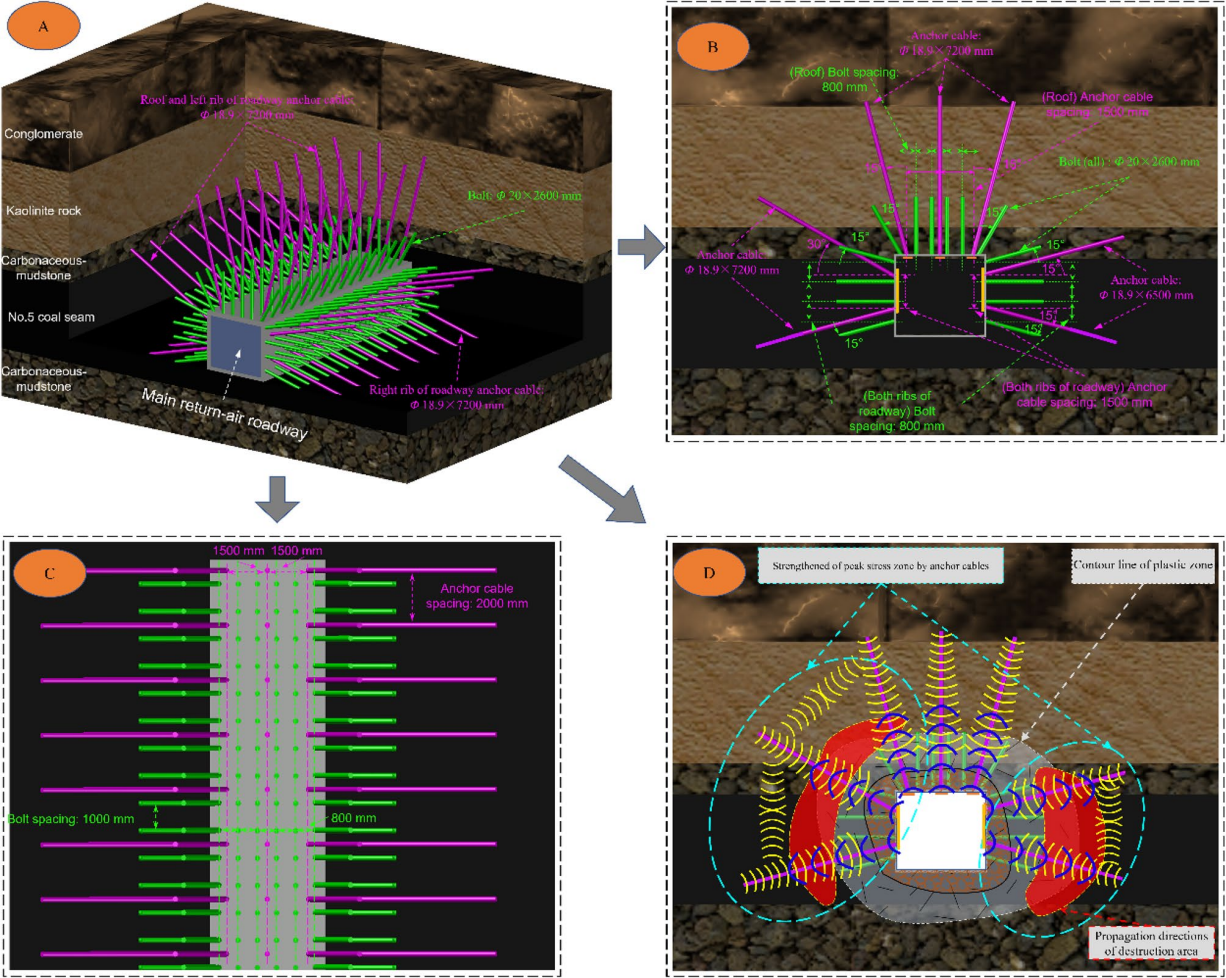
Reinforcement support scheme of No. 5 coal seam’s main return-air roadway (A: roadway support in the overall view; B: Front view of roadway support C: roadway support in the view of roof D: Stress field of supporting structure).

Based on the principle of non-symmetric orientation cooperative anchoring, a support scheme is proposed for NO. 5 coal seam’s main return-air roadway. The roadway roof cross-section is arranged with three anchor cables; the specifications are Φ18.9 × 7200 mm, the spacing is 1500 × 2000 mm, the specification of the pallet is 300 × 300 × 16 mm, the specifications of anchor bolts is Φ20 × 2600 mm left-hand rebar steel bolts, the spacing is 800 × 1000 mm, the pallet uses the butterfly pallet which the specification is 150 × 150 × 10 mm. The shotcrete thickness on both sides and the roof is 100 mm, and the strength grade is C20. Based on the above-mentioned conventional scheme, two reinforcing anchor cables are added on each side of the roadway. Channel steel is used to connect each side of the anchor cables: at the ribs near the solid coal side, two specifications Φ18.9 × 7200 mm anchor cables are added, the anchor cable near the roof is at an angle of 30° to the horizon, and the anchor cable near the floor is at an angle of 15° to the horizon. Two specifications Φ18.9 × 6500 mm bolting cables are added to the side of the roadway near the mined-out area, and the two anchor cables are at an angle of 15° to the horizon. The spacing of anchor cables on both ribs is 1500 × 2000 mm. Figure 14 shows the specific support design parameters.

The shallow compressive stress core zone formed by the bolt and cable in the conventional ribs can prevent the broken surrounding rock from falling off. However, it can not effectively support the critical area of stress increase and deflection changes (peak zone of *J*_2_). D of Fig. 14 shows that a deep compressive stress zone is formed by reinforcing anchor rope in the surrounding rock after united support. Two groups of functional deep compressive stress areas are formed in the peak zone of *J*_2_, and a systematic reinforcing stress field network is formed. It can effectively cover the critical area of *J*_2_ and transfer the mining stress from the shallow to the deep elastic rock mass to control the surrounding rock’s destruction.

### Field monitoring

Three measuring stations are placed along the working face in the mining roadway axis direction. Analysis of the mine pressure response of the roadway was carried out using the monitoring results. The interval of stations in each roadway is 15 m, and the main observation indexes are as follows: (1) advanced support pressure of mining roadway; (2) stress of mining roadway and main roadway anchor bolts and cables. Through the comprehensive diagnosis of the changing trend of the above indicators, combined with the stress field’s state under the different protective coal pillar widths in the No.5 coal seam, verify the reasonableness of the width of the coal pillar.

**Figure 15:**
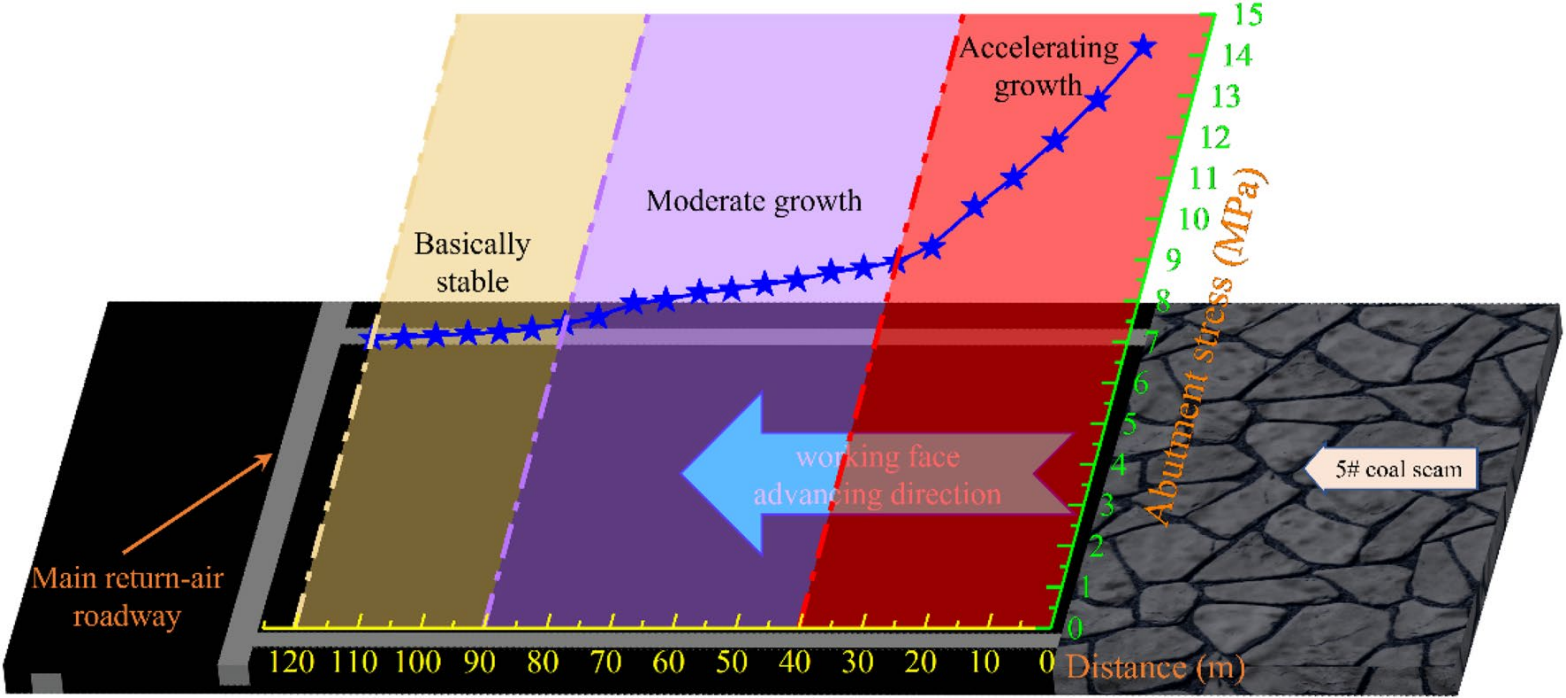
Monitoring result of the deep hole stress meter.

The borehole stress gauge measuring points are laid in the mining roadway of LWF7125. Each measuring point contains A and B, two stress gauges, respectively 14 m and 8 m deep into the coal, Fig. 15 shows the mining roadway abutment stress monitoring results.

During NO.5 coal seam mining: when the distance is greater than 90 m from the working face to the stress gauge, it is an insignificant fluctuation in stress gauge readings, and the state is the Overall stable trend. However, when the distance is less than 50 m from the working face to the stress gauge, the values increase sharply compared with the previous stage. With the mining of NO. 5 coal seam, the high-sensitivity borehole stress gauge approaches the working face, the advanced abutment pressure’s growth rate increases.

**Figure 16:**
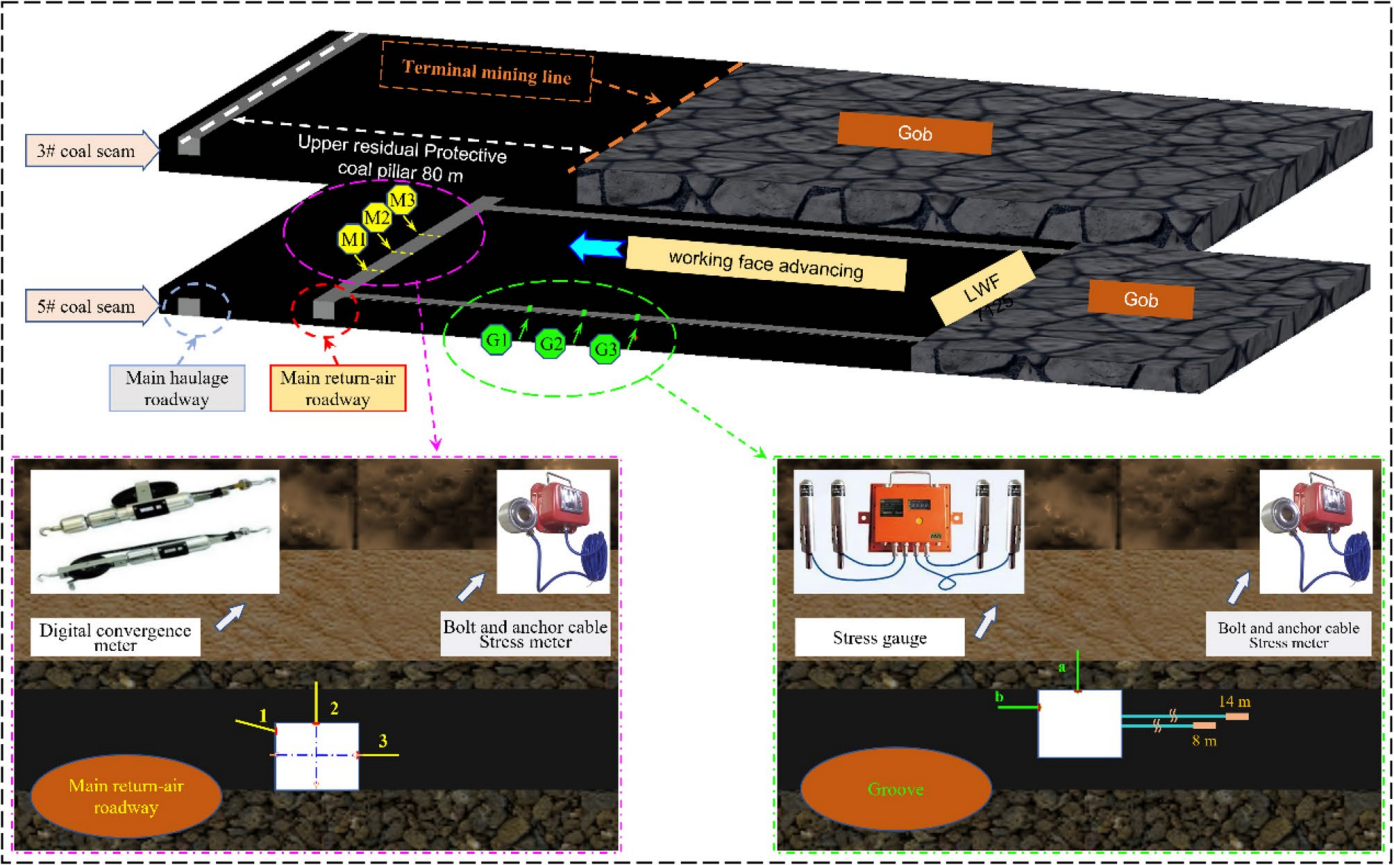
Principle of monitoring.

**Figure 17:**
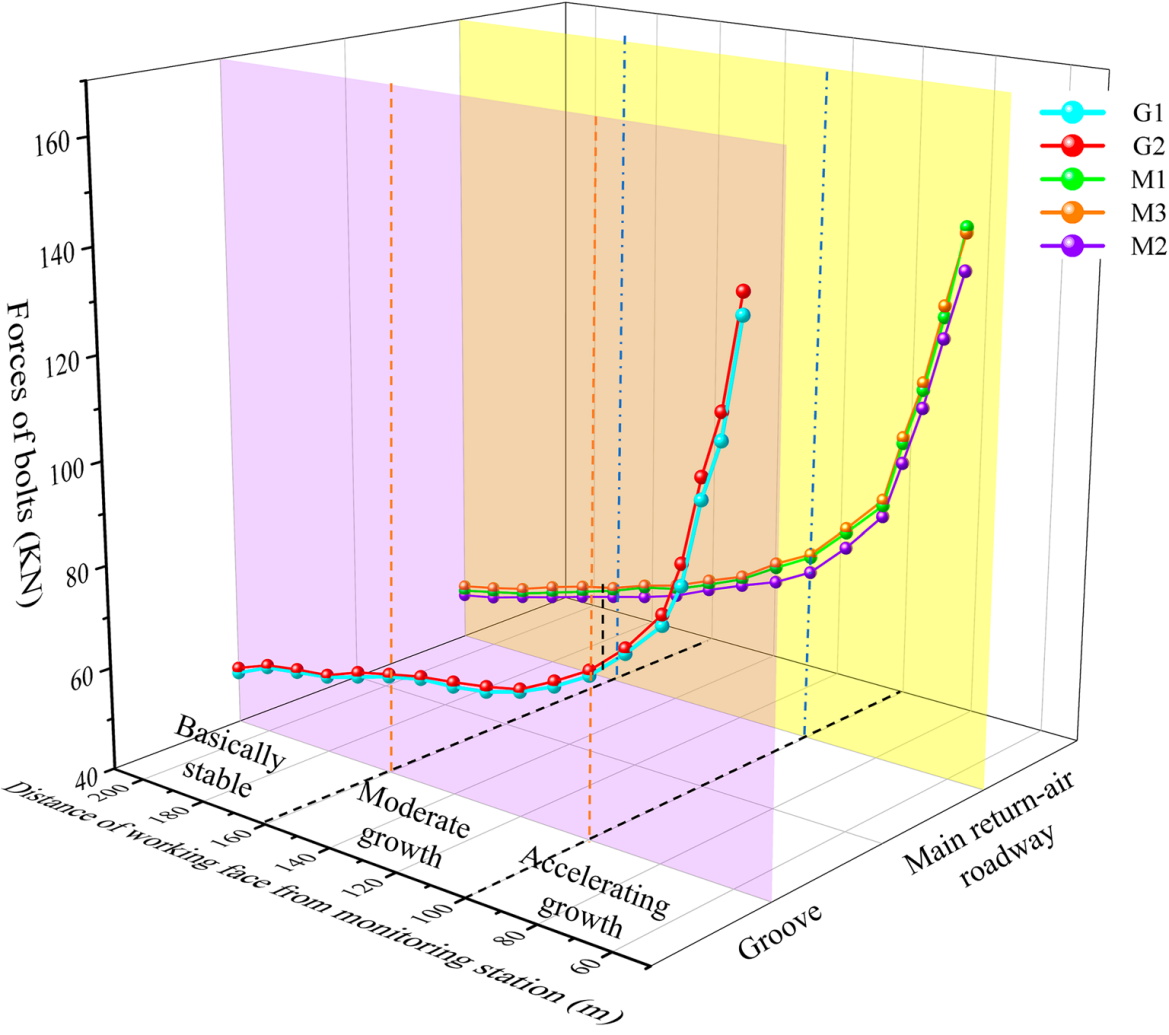
Monitoring results of the anchor bolt and cable stress meter.

Monitoring bolt-cable support resistance can reflect the effect range of mining and the surrounding rock deformation damage state in real time. Figure 16 shows the monitoring station arrangement, and Fig. 17 shows the monitoring results.

The pre-stress extent of the mining roadway's bolts of NO. 5 coal seam is 49–52 KN. After the mining of NO. 5 coal seam is stable, the stress meter readings are in the range of 141–145 KN, and the breaking load of the bolts is not reached (78.3–80.5%) during the mining. When the distance is greater than 100 m from the working face to the stress gauge, it is an insignificant fluctuation in stress gauge readings, and when the distance is from 100 to 40 m ahead of the working face to the stress gauge, the reading shows a slowly rising trend. The reading increases rapidly when the distance is less than 40 m from the working face to the stress gauge. This process also reflects that the 40 m ahead of the LWF7125 is most severely effect by mining, which remarkably increases the advanced abutment stress.

The pre-stress extent of the bolt in the main return-air roadway of NO. 5 coal seam is 48–53 KN. During the mining, the overall change trend of the stress meter readings is consistent with the stoping roadway. When NO. 5 coal seam set up the 60 m protective coal pillar, the stress meter readings of the main roadway’s two ribs are more significant than the roof, it shows a typical asymmetric characteristic. Comparing the readings of M1, M2, and M3, it is found that the two ribs of the roadway, especially the rib adjacent to the mined-out area, are more sensitive to the influence of mining. The field monitoring results agree with the critical area of stress increase and deflection changes. This shows that the theory is reasonable and can be applied to the deformation early warning and strengthening support of surrounding rock.

## Conclusion

In this paper, theoretical analysis is used to guide the numerical simulation experiments, and combined with the field monitoring results, the evolution model of the roadway surrounding rock’s *J*_2_ peak zone at different scales of stress increase and deflection changes is studied. The conclusions are as follows:

1. Under the superimposed influence of close double coal seam mining, the stress field of the main roadway shows typical asymmetric characteristics when lateral pressure coefficients are different, and the staggered distance between the two working faces is different. The principal stress direction deflects obviously with the mining and the deflecting degree and velocity increase as the greater of lateral pressure coefficient. At the same time, the peak stress field has an asymmetric distribution. The asymmetry becomes apparent with the decrease of NO. 5 coal seam’s protective coal pillar’s width.
2. The surrounding rock is affected by double-seam superimposed mining, resulting in two possible *J*_2_ peak zone development trends and three types of evolution models. When the scale of stress increases and deflection changes is weak, the *J*_2_ peak zone in the roadway surrounding rock develops in a double-wings pattern (two directions corresponding to the minimum principal stress), the *J*_2_ peak zone evolves in a four-wings pattern (going deep into the surrounding rock in four directions), which is not easy to support. There are three types of possible *J*_2_ peak area evolution models in the roadway surrounding rock with different scales of stress increase and deflection changes: two ribs area (the distribution of *J*_2_ peak zone along two sides of roadway), roof-floor area, and the roof-floor-ribs transfer area (2 kinds: upper left-lower right corner area, lower left-upper right corner area).
3. According to the development trend and evolution model of the critical area of stress increase and deflection changes of *J*_2_, the collaborative anchoring method of asymmetric truss-anchor cable is put forward. The core compressive stress network formed by the support structure can effectively cover the peak zone of *J*_2_ and then control the surrounding rock’s destruction and maintain roadway stability.
4. According to the simulation analysis and the field monitoring results, the following conclusions are drawn: after the upper coal seam is left with an 80 m protective coal pillar, the effect of mining is the most serious ahead 40 m of the lower coal seam working face; When a 60 m protective coal pillar is set in NO. 5 coal seam, the surrounding rock of the ribs is the key control zone of the stress increase and deflection changes, which is convenient to support while the resources are recovered. The phenomenon shows that it is reasonable and adequate to control the stability of surrounding rock using the collaborative anchoring method of asymmetric truss-anchor cable.

## Data Availability

All data generated or analysed during this study are included in this published article.
